# Measuring gene expression divergence: the distance to keep

**DOI:** 10.1186/1745-6150-5-51

**Published:** 2010-08-06

**Authors:** Galina Glazko, Arcady Mushegian

**Affiliations:** 1 Department of Biostatistics and Computational Biology, University of Rochester Medical Center, Rochester, NY 14642, USA; 2 Stowers Institute for Medical Research, 1000 E 50th St., Kansas City MO 64110, USA; 3 Department of Microbiology, Molecular Genetics, and Immunology, University of Kansas Medical Center, Kansas City, KS 66160, USA

## Abstract

**Background:**

Gene expression divergence is a phenotypic trait reflecting evolution of gene regulation and characterizing dissimilarity between species and between cells and tissues within the same species. Several distance measures, such as Euclidean and correlation-based distances have been proposed for measuring expression divergence.

**Results:**

We show that different distance measures identify different trends in gene expression patterns. When comparing orthologous genes in eight rat and human tissues, the Euclidean distance identified genes uniformly expressed in all tissues near the expression background as genes with the most conserved expression pattern. In contrast, correlation-based distance and generalized-average distance identified genes with concerted changes among homologous tissues as those most conserved. On the other hand, correlation-based distance, Euclidean distance and generalized-average distance highlight quite well the relatively high similarity of gene expression patterns in homologous tissues between species, compared to non-homologous tissues within species.

**Conclusions:**

Different trends exist in the high-dimensional numeric data, and to highlight a particular trend an appropriate distance measure needs to be chosen. The choice of the distance measure for measuring expression divergence can be dictated by the expression patterns that are of interest in a particular study.

**Reviewers:**

This article was reviewed by Mikhail Gelfand, Eugene Koonin and Subhajyoti De (nominated by Sarah Teichmann).

## Background

The genome-wide data often take the form of a series of measurements associated with every gene in the genome. This series of numbers have been called 'gene vectors', and many investigations in comparative genomics and systems biology start with determining distances, or similarities/dissimilarities, between all pairs of gene vectors in the measurement space, in order to use these distances for discovery of relationships between genes [1]. In the context of genome-scale gene expression measurements, the subject of this study, one of the simplest and most important kinds of such relationships may be co-expression of genes - for example, similar pattern of expression values of two genes across the time course of the experiment, or across different tissues of the same organism, or similar pattern of expression values of two orthologous genes in homologous tissues of related organisms [2].

Many mathematical formulations are available for distances between two vectors, and it is of interest to know how to choose the appropriate distance measure among many. Much of previous work on distance measures in computational biology focused on such properties as metric and additive [3,4], which have a close connection to the computational tractability of the clustering algorithms, but, generally, are not designed to tell anything about the biological plausibility of the groups of genes generated by any given combination of measure and algorithm. There is no general solution to the problem of choosing optimal distance measure for any kind of genome-scale data, and the choice has to be guided by the additional information about the data, e.g., the knowledge of the data-generating process model or the existence of a benchmark dataset.

The question of comparing gene expression profiles arises in the functional context (i.e., which genes tend to be regulated together?) and in the evolutionary context. The patterns of gene expression are inheritable traits, and several models of evolution of expression have been proposed [5-7]. Despite this work, and even some success in inferring the ancestral state of gene expression [8], the methods for estimating the evolutionary distance (divergence) between genes and species from their gene expression profiles is in its infancy. To estimate expression divergence between different species, a variety of measures has been used, such as Euclidean distance, correlation-based dissimilarity and other distances (e.g. [2,9-12]), but little was known about relative advantages of each measure.

In a recent study Pereira et al [13] investigated the choice of the distance measure between gene expression profiles across different tissues for human, mouse and rat. In their approach, there were three sets of expression profiles, one for each species, and each gene vector had eight coordinates (tissue samples in which gene expression levels were measured). The one-to-one orthologous relationships [14,15] exist between a large fraction of genes in three species, so distances between pairs of orthologous gene vectors from each of the three possible pairs of species can be examined. It has been found that the correlation-based distance overestimates the expression divergence for genes with approximately uniform expression patterns between different tissues in the three species, probably because of the random noise that is uncorrelated between species. This effect was not observed with Euclidean distance. Moreover, the two measures of expression difference between orthologous genes were largely uncorrelated between all pairs of species. It has been concluded that Euclidean distance has the advantage of not amplifying the noise. Additionally, Euclidean distance was stated to be more sensitive to the absolute level of gene expression than correlation-based distance.

In this work, we explore the theme of the optimal choice of distances for analysis of gene expression profiles and make a case for a close fit between the mathematical properties of the distance measure and the biological question at hand. The choice of distance should be informed by the properties and signals in the data that are of interest in a particular study: the measure that is best suited to detect and highlight these signals will be optimal in the context of that study.

## Results and Discussion

### Empirical criteria for distance performance

When expression of orthologous genes across homologous tissues is to be compared between species, biological sensibilities suggest two trends. First, we expect homologous tissues between species (e.g. rat kidney and human kidney) to be more similar on average than non-homologous tissues within the same species (e.g. rat kidney and rat skeletal muscle) [2]. Second, we expect that evolution of gene expression is constrained [2,9], *i.e*., expression divergence between orthologous gene pairs in two species is on average significantly lower than between random gene pairs, one each from the same two species. These two well-defined properties may be used as empirical criteria for selecting the best-performing distance. First, a good distance measure will cluster tissues rather than species: if we cluster samples in the gene space, the homologous tissues of human and rat will tend to have each other, not the same-species tissues, as their nearest neighbors in the cluster. Second, a good distance measure should cluster the orthologous genes in excess over random genes.

We evaluated the Euclidean distance and correlation-based distance in these tests. In addition, we also clustered the data using generalized average, a parametric family of distance measures applicable to the special case of binary gene vectors. The notable properties of this family is that it includes a large variety of known distance measures as special cases, and that an empirical statistical criterion of selecting well-performing distance measures, independent of the biological considerations mentioned above, has been given [1].

### Data transformation approaches

We downloaded raw CEL files for human and rat from Gene Expression Omnibus (GSE2361 [16], GSE952 [17]). There were 3152 one-to-one orthologous gene pairs (Ensembl, release 57, BioMart) in eight tissues (bone marrow, heart, kidney, pituitary, skeletal muscle, small intestine, spleen, and thymus) simultaneously present in human and rat data (see Methods for detail). Expression data can not be compared across species directly. For example, in our data set the distribution of gene expression intensities for humans is shifted to the right compared to the distribution of gene expression intensities for rats (Additional File 1, Figure S1). Without a correction, we would see about 1200 orthologs as significantly differentially expressed. To avoid "discoveries" of this kind, various transformations have been proposed, for example the relative expression of Liao and Zhang [2]: if *x^A^_ij _*is the absolute expression level of gene *i *in tissue *j *then the relative expression *x^R^_ij _*of gene *i *is $x^{R}{}_{ij}=x^{A}{}_{ij}/\sum_{j=1}^{n}x^{A}{}_{ij}$, where *n *is the number of tissues. We computed two distances between relative expression vectors: the Euclidean distance, $d_{E}=\sqrt{\sum_{j=1}^{n}{\left(x^{R}{}_{hj}-x^{R}{}_{rj}\right)}^{2}}$ and correlation-based distance, *d_cor _*= 1-*r*(*x^R^_h_*, *x^R^_r_*), where *x^R^_h, _x^R^_r _*are relative expression levels for any gene in human and rat, respectively and *r *stands for the Pearson correlation coefficient.

Another data transformation method frequently applied in gene expression studies is the binary transformation [18]. In our data set there could be at least two different patterns of gene expression. In the first pattern, if a gene is tissue-specific and is expressed in one particular tissue, then its expression is much higher than the average for a given gene over all tissues. In the second pattern, a housekeeping gene is expressed more or less consistently across all tissues. We therefore applied simple binary transformation: *x^B^_ij _*= 1 if $x^{A}{}_{ij}\geq {\bar{x}}^{A}{}_{i}$ and *x^B^_ij _*= 0 otherwise. Then coordinates of a binary transformed vector corresponding to tissue-specific gene will be mostly zeros with infrequent ones, while those for a housekeeping gene will be almost all ones with only occasional zeros. This transformation allows us to use generalized-average (GA) distance measure [1]. For a pair of binary vectors, *x^B^_m _*and *x^B^_n_*, (here *m *and *n *indicate the total number of ones in a vector) GA distance is calculated as *d_Aλ, mn _*= 1-*A_λ, mn_*, where $A_{\lambda ,}{}_{mn}=\frac{X_{mn}}{B_{\lambda }}$ , (-∞ < λ < ∞), *X_mn _*= *x^B^_m_x^B^_n _*is the scalar product of two vectors and $B_{\lambda }={\left(\frac{X^{\lambda }{}_{mm}+X^{\lambda }{}_{nn}}{2}\right)}^{\frac{1}{\lambda }}$ is the generalized average cardinality of two vectors, of exponent λ. From this expression one can obtain distances based on the Simpson similarity index (λ → -∞) or Dice similarity index (λ = 1) related to Jaccard similarity index, as well as many others (see [1] for detail). For the binary-transformed data set we also applied correlation-based distance, *d^B^_cor._*

### Distance estimates

First, we calculated expression divergence between human and rat homologous tissues. To select best performing GA distances we employed the empirical criterion suggested in [1], namely that the distribution of best performing distance tends to have the extreme values of third and forth moments. We therefore calculated GA for several lambda parameters (Additional File 1, Figure S2a) and found that GA distance with λ → ∞ had the lowest value for skewness and the distance based on correlations for binary transformed gene expressions had the lowest values for kurtosis (Additional File 2, Figure S2a). We decided to test both of these distances, in addition to conventional correlation-based distance and the Euclidean distance, on the interval coordinates.

**Figure 1:**
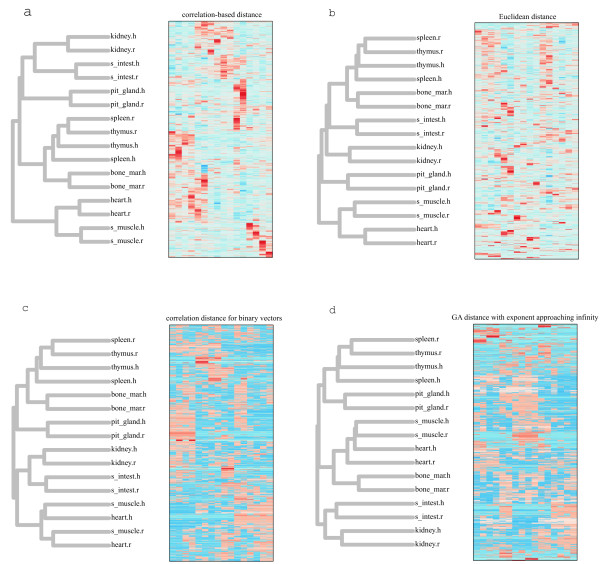
**Clustering of human and rat tissues and genes**. Clustering is based on **(a) **correlation-based, **(b) **Euclidean, **(c) **binary correlation distances and **(d) **generalized-average distance with exponent approaching infinity.

Figure 1 presents clustering of human and rat tissues and genes based on different distances. Correlation-based distance and the Euclidean distances cluster six out of eight tissues correctly (Figure 1a, and Figure 1b), despite small sample size, and distance measures for binary transformed expression values cluster four tissues (Figure 1c and Figure 1d), indicating some loss of signal due to discretization. Nonetheless, it is clear that the correlation-based distance and the Euclidean distance are performing equally well in the problem of tissue clustering, and even for the binary transformed data the correlation-based distance detects some of the relevant signal.

**Figure 2:**
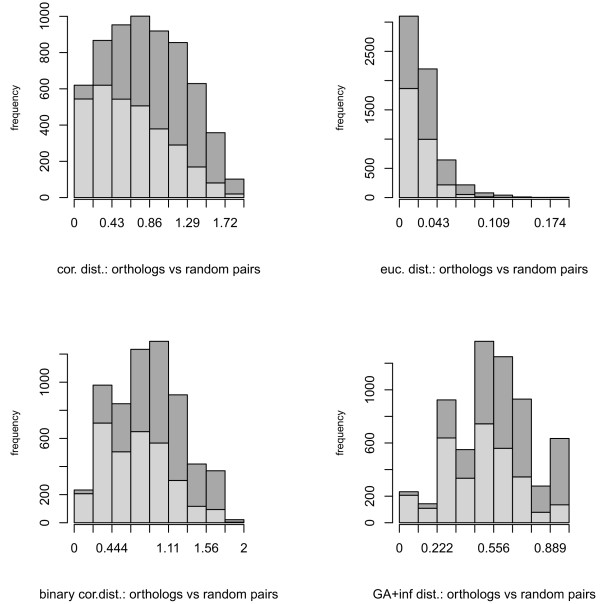
**Histograms of distances between orthologous and random gene pairs**. The histogram bars corresponding to orthologous and random pairs are shown in light and dark gray, respectively.

Next, we calculated expression divergence between human-rat orthologous gene pairs and between human-rat random gene pairs using different distances. Again, to select best performing GA distances, distribution statistics for several lambda parameters (Additional File 2, Figure S2b) were computed. In this case distribution statistics were less variable, but GA distance with λ → ∞ had the lowest skewness and the distance based on correlations for binary transformed gene expressions had the lowest skewness and the highest kurtosis (Additional File 2, Figure S2b); again we decided to try both of them. Distance distributions between 3152 orthologs and 3152 random pairs of human-rat genes were constructed for four different distances (Figure 2). At the 1% significance level correlation-based distance, the Euclidean distance, binary correlation-based distances and GA distance (λ → ∞) identified, respectively, 327, 69, 207 and 215 orthologous gene pairs with conserved expression profiles.

**Figure 3:**
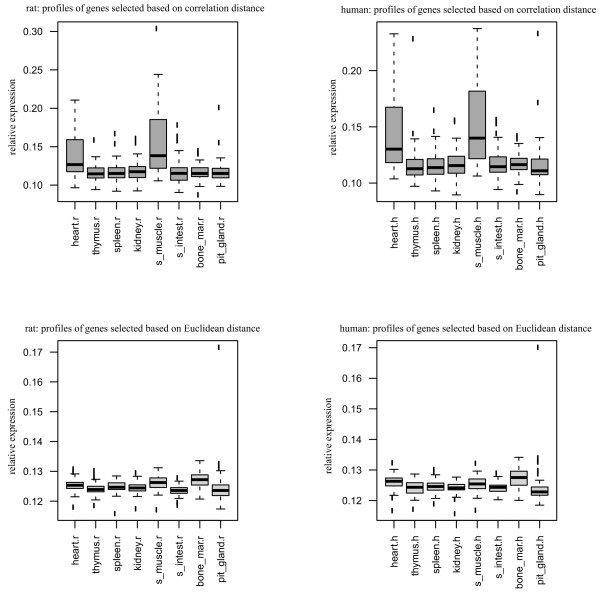
**Expression profiles of conserved orthologous gene pairs in two species over eight tissues**. Upper panel: expression profiles selected using correlation-based distance. Lower panel: expression profiles selected using the Euclidean distance.

To better understand the differences between the four distances, we analyzed functional enrichment of the identified conserved gene pairs. For the four groups of genes we calculated overrepresented GO terms (*p*-values≤ 0.001) using GOstat [19,20]. Genes identified using correlation-based distance, binary correlation distance, and GA distance shared 15 overrepresented GO categories (Additional File 4, Table S1), whereas genes identified using the Euclidean distance were from a broad variety of different GO categories (Additional File 4, Table S2). Genes identified with three former distances tend to belong to biological processes involved in muscle and heart development and morphogenesis, while genes identified with the Euclidean distance represent a different processes. Expression profiles of genes from Table S1 and Table S2 (Additional File 4) shed more light on the nature of differences between distances (Figure 3). As one can see (Figure 3, lower panel), for both species genes selected using the Euclidean distance tend to be expressed in all tissues at the uniformly low level, close to the background. In contrast, genes selected using correlation-based distance tend to be expressed in several homologous tissues (Figure 3, upper panel) at the much higher level. That is, the expression conservation found with the Euclidean distances tends to come from genes with low expression and without a single major theme in biological processes in which these genes are involved (Additional File 4, Table S2). This suggests an interpretation of the difference between correlation-based distances and the Euclidean distances noted in [2]. The Euclidean distance measures the uniform divergence between expression profiles, the higher the divergence the larger the distance is. The correlation-based distance measures the concerted changes between profiles: the less changes profiles share, the larger the distance is.

### How high entropy scales with uniform expression

Pereira et al [13] defined uniformly expressed genes as genes that have high entropy. This definition came from the observation that gene expression entropy changes from 0 for genes expressed in just one sample to log_2_(*n*) for genes expressed in all *n *samples [21]. They selected genes with the entropy from the upper quartile of the gene expression entropy distribution, expecting that 'genes with a conserved uniform pattern of expression' will have low pattern of expression divergence, but observed exactly the opposite using correlation-based distances. Namely, genes with high entropy had higher correlation-based distances than other genes; in contrast the Euclidean distances between them were low.

Following this procedure we identified 788 genes in the upper quartile of the entropy distribution. Expression profiles for these 788 genes across eight different tissues in two species, as well as for 788 randomly selected genes clearly show that genes with high entropy are not 'genes with a conserved uniform pattern of expression' (Additional File 3, Figure S3). The requirement of high entropy indeed selects genes that are expressed in all tissues (Figure S3, upper panel, Additional File 3), in contrast to the rest of the genes (Additional File 3, Figure S3, lower panel). However, the variance term does not enter into the formula for calculating entropy, so genes selected on the basis of high entropy often have highly variable expression. In addition, genes with high entropy seem not to vary in concert across tissues in two species (Additional File 3, Figure S3, upper left and right panels). The variance term is included in correlation-based distance and the less concerted are changes between genes, the larger is correlation-based distance. In contrast, the Euclidean distance does not include the variance term and will give an impression of low divergence between genes with high entropy, especially when the absolute expression level is low. Thus, one could say the observed pattern of expression divergence measured by correlation-based distance is not a shortcoming of the distance, but a shortcoming of the definition of the 'uniform pattern of expression' when uniformity is understood as high entropy.

## Conclusions

We would like to emphasize that different trends exist in the high-dimensional numeric data, and different distance measures highlight differently some of these trends. In the present case, three types of distances highlighted relatively well the property of gene expression profiles to be more similar for homologous tissues between species than for non-homologous tissues within species. In contrast, when answering a question about divergence between orthologous genes, one of the three distances (Euclidean) selected genes uniformly expressed in all tissues near the expression background, while correlation-based distances and GA distance selected genes with concerted changes among homologous tissues. Thus, for studying the expression divergence in different species the choice of the distance measure has to be guided by the kind of the expression patterns one would like to identify.

## Methods

Raw CEL files for human and rat were downloaded from Gene Expression Omnibus (GSE2361 [16], GSE952 [17]). Similar to [13] we selected eight tissues simultaneously present in human and rat data: bone marrow, heart, kidney, small intestine, pituitary gland, skeletal muscle, spleen and thymus. There were two arrays for rat tissues and only one array for humans, so we averaged rat expression values across tissue replicates. Raw data were normalized using RMA procedure [22]. When multiple probe sets per gene were available we selected the one with the largest value of the overall expression. Human-rat gene pairs annotated as having a one-to-one orthologous relationship (3152 pairs) were obtained with Ensembl, release 57, BioMart.

## Competing interests

The authors declare that they have no competing interests.

## Authors' contributions

GG designed the study and analyzed the data. AM suggested the theme. Both authors wrote the manuscript. All authors read and approved the final manuscript.

## Reviewers' comments

*Reviewer 1: Mikhail Gelfand, Department of Bioengineering and Bioinformatics, Moscow State University, and Institute for Information Transmission Problems RAS, Moscow, Russia*

The paper addresses an important problem of selecting a good similarity measure for comparing gene expression patterns. It does not provide definitive answers, but demonstrates correct approaches. The main conclusion, "the choice of a proper measure depends on the biological problem at hand" is difficult to argue against. The following comments are mainly of the discussion and editorial nature.

While the basic assumption, that homologous tissues in different organisms should be more similar in the terms of gene expression than tissues in one organism, is reasonable, some caveats are due. For instance, if the tissues in question are very close developmentally, one can easily expect concerted, organism-specific changes in expression. In fact, the papers results demonstrate exactly that.

The rat spleen and thymus are clustered by all measures (Fig. 1). The human spleen and thymus are clustered by some measures, and I think that clustering [(thymus_rat + spleen_rat) + (thymus_human + spleen_human)] should not be counted as an error, as opposed to a version with human spleen being an outlier: [((thymus_rat + spleen_rat) + thymus_human) + spleen_human]. Similarly, I'd assume that both versions [(muscle_human + heart_human) + (muscle_rat + heart_rat)] and [(muscle_human + muscle_rat) + (heart_human + heart_rat)] are biologically relevant, as opposed to [((muscle_human + heart_human) + muscle_rat) + heart_rat)]. Hence, the procedure of counting errors should not be limited to considering pairs of non-clustered homologous tissues, but should tale into account finer topological detail (as well as, maybe, branch length).

*Authors' response*: We agree with the reviewer that there may be more than one biologically relevant clustering solution, and concerted organism-specific co-expression of genes might cause species-specific tissue cluster. However, we believe that in most cases non-homologous tissues clustering is directly related to tissues sampling and the number of replicates available. Curiously, the pattern [((thymus_rat + spleen_rat) + thymus_human) + spleen_human], was observed with all four distance measures that we tried. Also note that part of our intention was to demonstrate that in the problem of tissue clustering there is no valid reason to dismiss the correlation-based distance, despite the concerns raised in ref. [13]; and indeed, correlation-based distance and the Euclidean distances gave the same results in our hands, and even for the binary transformed data the correlation-based distance detected some of the relevant signal.

While this may go beyond the limits of the present study, I think it would be interesting to look into more detail into the cluster trees generated by different measures, and specifically, into what genes contribute most into different clusters, dependent on the expression patterns. At that, one should keep in mind that in each tissue we observe an averaged expression of genes from a mixture of quite different cell types. For instance, clustering of the spleen, thymus and the bone marrow may be related to the blood cells development, while clustering of the spleen, thymus and the pituitary gland may be caused by genes expressed in the gland tissue.

Some hint of analysis is given in the last paragraph of "Distance estimates". The overrepresentation of heart and muscle development genes is not surprising, given the robust clustering of these tissues in all trees. On the other hand, the statement that the Eucledian distance does not provide a functionally meaningful set: one can easily see blood cell development genes there (not surprising given spleen, thymus and bone marrow data) and neurological process (the sources for which is admittedly less clear: could it be the pituitary gland?)

*Authors' response*: We agree that there is good information in the clusters produced by Euclidean distance, even if there is no single dominant theme there. Note, however, that genes selected using the Euclidean distance tend to be expressed in all tissues at the uniform low level, while genes selected using correlation-based distance tend to be expressed in several orthologous tissues at the much higher level.

*Reviewer 2: Eugene Koonin, National Center for Biotechnology Information, National Library of Medicine, National Institutes of Health*

The paper by Glazko and Mushegian makes the case that different measures of expression divergence (in particular, Euclidean distances and correlation-based distances) are best suited for revealing different trends in the evolution of gene expression. I would like to strongly endorse this work that shows flexibility which is vital for understanding such a complex phenomenon as evolution of gene expression in multicellular organisms. A versatile approach like this gives the only hope of progress in this field and is a welcome contrast to the common attempts to propose one approach claimed to be best for all purposes.

*Authors' response*: We appreciate the reviewer positive comment. Taking a more familiar example of distances between biological sequences, we know that those can be roughly estimated even without an explicit model of sequence evolution, but it is also known that, as sequences diverge, the error of the estimate becomes more and more significant. Similarly, the ultimate goal in gene expression analysis is to have an evolutionary model for gene expression. Short of that, the divergence between expression profiles can be estimated with appropriate distance measures.

*Reviewer 3: Subhajyoti De (nominated by Sarah Teichmann), Computational Biology Program, Memorial Sloan-Kettering Cancer Center*

In the paper entitled "Measuring gene expression divergence: the distance to keep", Glazko and Mushegian present a discussion about which distance measure to use in inter-species expression divergence analyses. While the topic is of broad interest, I have some comments

Major comments

1. How were the transcripts with multiple probes treated? How were the probes that map to multiple genes treated?

*Authors' response*: Raw data preprocessing step is described in the Method section.

If a gene had multiple transcripts, how did the authors choose the representative transcript?

*Authors' response*: Affymetrix Human hgu133a and Rat rgu34a arrays do not provide information about multiple transcripts.

Why no between-array normalization was performed for rat samples?

*Authors' response*: RMA procedure was implemented for both human and rat arrays.

2. The distributions of Euclidean distance and correlation-based distance for pairs of randomly chosen gene pairs differ in their shapes. Can the authors discuss this issue and also how that may affect their comparative analysis and tree-building?

*Authors' response*: This is exactly the point of the presented paper. Not only the distributions between randomly chosen gene pairs are different, but also the distributions between orthologous gene pairs are different for all distance measures that we tried. As we have shown in the paper, this difference most certainly may have an effect on the analysis, and the kind of effect depends on the type of the analysis, i.e., on the biological question that is asked.

3. In the recent releases of Ensembl, there are about 14,000 one-to-one orthologs. The authors present results based on 3152 genes. It remains to be clear why the dataset analyzed is so small and whether the conclusions made in this paper can be extended to the whole genome dataset.

*Authors' response*: hgu133a and rgu34a arrays contain 22283 and 8799 probe sets, respectively. After mapping them to unique genes, only 4939 genes for rat were left. The conclusions made in this paper refer to the distance properties and hardly depend on the number of the orthologs studied.

4. In Figure 1 it is not clear how the tree was drawn (e.g. Neighbour joining, Maximum likelihood) and how that method may affect the tree structure. Furthermore, the authors should perform bootstrapping to assess the quality of the trees.

*Authors' response*: We used average-link clustering for tree inference. As we were interested in how different distance measures affect the tree structure, we applied the same clustering approach to each distance matrix. Different clustering approach may indeed produce trees with different topologies, but we expect that the effect of varying distance measure would be observed in any clustering algorithm. As for the support of the trees, we expect it to be relatively low given the sample size and the amount of replicates, and our focus here is on the qualitative estimate of how different distances perform in the problem of tissues clustering.

5. In Figure 2 the histogram bars corresponding to orthologus and random gene pairs should be provided side-by-side. In its current form, it is hard to interpret how the distributions of orthologus gene-pairs differ from the random pairs.

*Authors' response*: We think that bar plots with stacked columns demonstrate the difference between these distributions quite clearly.

6. In Figure 3, y-axis label is missing. Why skeletal muscle shows high Euclidian and correlation distance that is significantly above other tissue-types (as seen by boxplot) and the trend is consistent in all the four panels? Is it an array normalization artifact or a biologically meaningful pattern?

*Authors' response*: We labeled y-axis in Figure 3. The meaning of the pattern observed in Figure 3, we believe, is that genes selected using the Euclidean distance tend to be expressed in all tissues at the uniformly low level (close to the background), while genes selected using correlation-based distance tend to be expressed in several orthologous tissues at a higher level.

Minor comments:

1. The Ensembl Release version is not provided.

*Authors' response*: The release version is now included.

2. GO has many functional categories organized in a hierarchical structure. It is unclear which level of GO hierarchy was used in the current analysis.

*Authors' response*: The levels were chosen based on the significant *p*-values provided by the enrichment test, and therefore the categories from different levels of the hierarchy could be reported.

3. Table S1 and S2 carry insufficient detail about the methodology involved and the message they convey. For instance, it is unclear whether the over-represented GO categories in Table S1 arise from analysis on heart tissue? How is the p-value calculated?

*Authors' response*: We now provide more comprehensive description of Tables S1 and S2 in Additional file 4. We first identified orthologous gene pairs with expression profiles conserved at the 1% significance level, using different distances. For these gene pairs we implemented GO enrichment analysis. Genes identified using correlation-based distance, binary correlation distance, and GA distances shared 15 overrepresented GO categories (Table S1), whereas genes identified using the Euclidean distance were from completely different GO categories (Table S2). This was the lesson learned from the analysis, i.e., that different distances select functionally different conserved orthologous gene pairs. The over-represented GO categories in Table S1 arise from the genes expressed in all tissues and identified as conserved by three different distances. *p*-values were calculated by hypergeometric test using the GOstat module from Bioconductor.

4. In Figure S3, in each panel, the outliers cross the whisker and also appear to be shifted. Please revise the figure. Also please adjust the y-axis scale in the two bottom panels to make the figures easier to visualize.

*Authors' response*: In R implementation, whiskers extend to 1.5*IQR but the parameters can be adjusted so that outliers are not displayed at all. The message of Figure S3 is that genes with high entropy are not 'genes with a conserved uniform pattern of expression'.

## Supplementary Material

Additional file 1 **Supplementary Figure S1: The distributions of gene expression intensities and MASS *p-*values for human and rat**. Click here for file

Additional file 2 **Supplementary Figure S2: Statistics of distribution of GA-based distances with different exponents**. Click here for file

Additional file 4 **Supplementary Tables S1-S2**. Click here for file

Additional file 3 **Supplementary Figure S3: Expression profiles of genes with the entropy in the upper quartile of mean entropy values and randomly selected genes**. Click here for file
