# Supplementary material for: Measuring gene expression divergence: the distance to keep
Source: Biol Direct. 2010 Aug 6;5:51. doi: 10.1186/1745-6150-5-51 (PMC2928186; doi:10.1186/1745-6150-5-51)
Supplement: Additional file 1 — Supplementary Figure S1: The distributions of gene expression intensities and MASS p-values for human and rat. [file 1745-6150-5-51-S1.PDF]

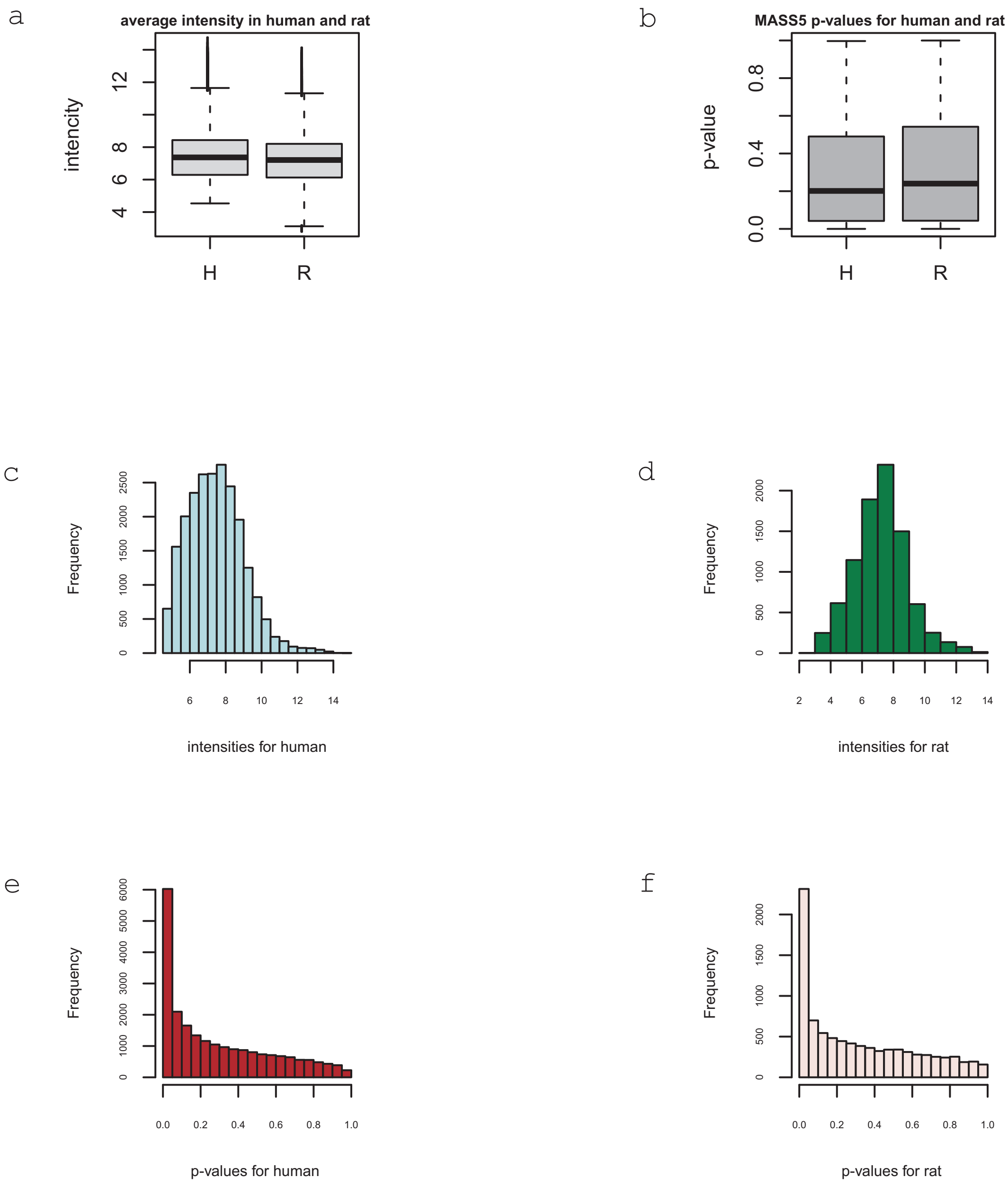

Figure S1. (a) Boxplots of the distribution of gene expression intensities for human (H) and rat (R). (b) Boxplots of the distribution of MASS *p*-values for human (H) and rat (R). (c) The distribution of gene expression intensities for human. (d) The distribution of gene expression intensities for rat. (e) The distribution of MASS *p*-values for human. (f) The distribution of MASS *p*-values for rat.
