# Supplementary material for: Measuring gene expression divergence: the distance to keep
Source: Biol Direct. 2010 Aug 6;5:51. doi: 10.1186/1745-6150-5-51 (PMC2928186; doi:10.1186/1745-6150-5-51)
Supplement: Additional file 2 — Supplementary Figure S2: Statistics of distribution of GA-based distances with different exponents. [file 1745-6150-5-51-S2.PDF]

a

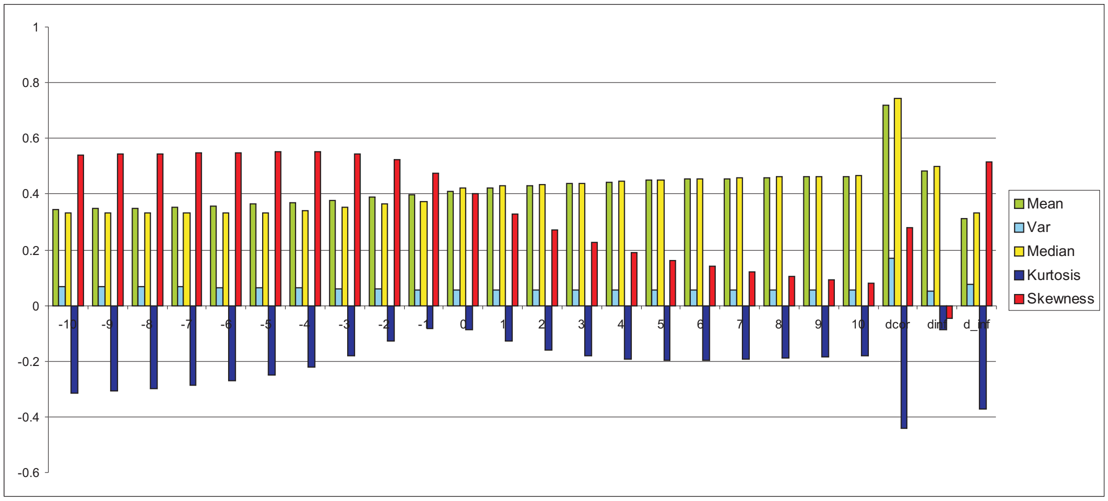

b

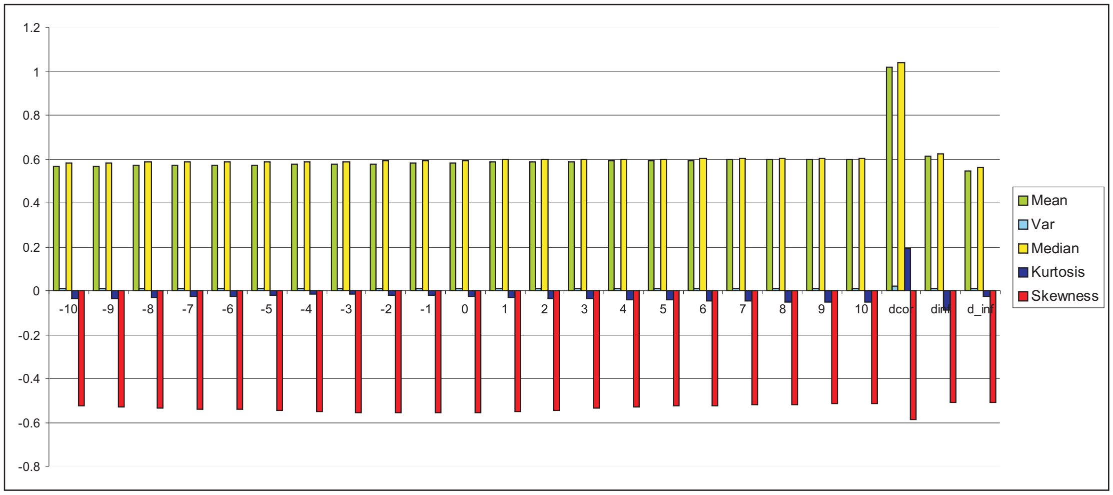

Figure S2. Statistics of distribution of GA-based distances with different exponents (varying from -10 to 10 and approaching minus and plus infinities) and correlation-based distance for binary transformed gene expressions for (a) tissue clustering and (b) orthologous gene pairs.
