# Supplementary material for: Measuring gene expression divergence: the distance to keep
Source: Biol Direct. 2010 Aug 6;5:51. doi: 10.1186/1745-6150-5-51 (PMC2928186; doi:10.1186/1745-6150-5-51)
Supplement: Additional file 4 — Supplementary Tables S1-S2. [file 1745-6150-5-51-S4.PDF]

SUPPLEMENTARY TABLES

NB. We first calculated expression divergence between human-rat orthologous gene pairs and between human-rat random gene pairs using four distances; next we selected conserved orthologous gene pairs at the 1% significance level. For these gene pairs we implemented GO enrichment analysis in GOstat (Bioconductor). Genes identified using correlation-based distance, binary correlation distance, and GA distances shared 15 overrepresented GO categories (Table S1), whereas genes identified using the Euclidean distance were from completely different GO categories (Table S2). Conclusion: different distances select functionally different conserved orthologous gene pairs.

Table S1. The intersection of overrepresented GO categories for conserved orthologous gene pairs identified using correlation-base, GA and binary correlation distances.

GOBPID	Pvalue	OddsRatio	ExpCount	Count	Size	Term
GO:0006941	0.000	10.8	1.92	12	28	striated muscle contraction
GO:0003012	0.000	4.5	6.79	23	99	muscle system process
GO:0030048	0.000	21.0	0.69	6	10	actin filament-based movement
GO:0055010	0.000	17.4	0.62	5	9	ventricular cardiac muscle morphogenesis
GO:0060415	0.000	13.9	0.69	5	10	muscle tissue morphogenesis
GO:0060047	0.000	4.3	2.95	10	43	heart contraction
GO:0000272	0.002	11.1	0.62	4	9	polysaccharide catabolic process
GO:0002026	0.002	11.1	0.62	4	9	regulation of the force of heart contraction
GO:0007512	0.002	20.7	0.34	3	5	adult heart development
GO:0003013	0.004	2.3	7.89	16	115	circulatory system process
GO:0006091	0.004	2.4	7.20	15	105	generation of precursor metabolites and energy
GO:0043462	0.005	13.9	0.41	3	6	regulation of ATPase activity
GO:0007517	0.005	2.5	5.97	13	87	muscle organ development
GO:0003007	0.005	4.4	1.71	6	25	heart morphogenesis
GO:0009150	0.009	3.4	2.47	7	36	purine ribonucleotide metabolic process

Table S2. The overrepresented GO categories for conserved orthologous gene pairs identified using the Euclidean distance.

GOBPID	Pvalue	OddsRatio	ExpCount	Count	Size	Term
GO:0007268	0.000	4.8	3.73	14	168	synaptic transmission
GO:0050877	0.000	3.5	6.68	18	301	neurological system process
GO:0014059	0.000	34.6	0.16	3	7	regulation of dopamine secretion
GO:0007154	0.001	5.1	2.06	8	124	cell communication
GO:0050432	0.001	23.1	0.20	3	9	catecholamine secretion
GO:0015872	0.003	13.8	0.29	3	13	dopamine transport
GO:0045620	0.003	45.5	0.09	2	4	negative regulation of lymphocyte differentiation
GO:0006811	0.003	2.6	6.36	14	287	ion transport
GO:0007165	0.004	2.0	22.33	33	1007	signal transduction
GO:0051952	0.005	10.6	0.35	3	16	regulation of amine transport
GO:0007186	0.005	2.6	5.28	12	238	G-protein coupled receptor protein signaling pathway
GO:0048609	0.006	6.2	0.75	4	34	reproductive process in a multicellular organism
GO:0045577	0.007	22.7	0.13	2	6	regulation of B cell differentiation
GO:0022602	0.009	8.1	0.44	3	20	ovulation cycle process
GO:0020027	0.009	18.2	0.16	2	7	hemoglobin metabolic process
