# Supplementary material for: Measuring gene expression divergence: the distance to keep
Source: Biol Direct. 2010 Aug 6;5:51. doi: 10.1186/1745-6150-5-51 (PMC2928186; doi:10.1186/1745-6150-5-51)
Supplement: Additional file 3 — Supplementary Figure S3: Expression profiles of genes with the entropy in the upper quartile of mean entropy values and randomly selected genes. [file 1745-6150-5-51-S3.PDF]

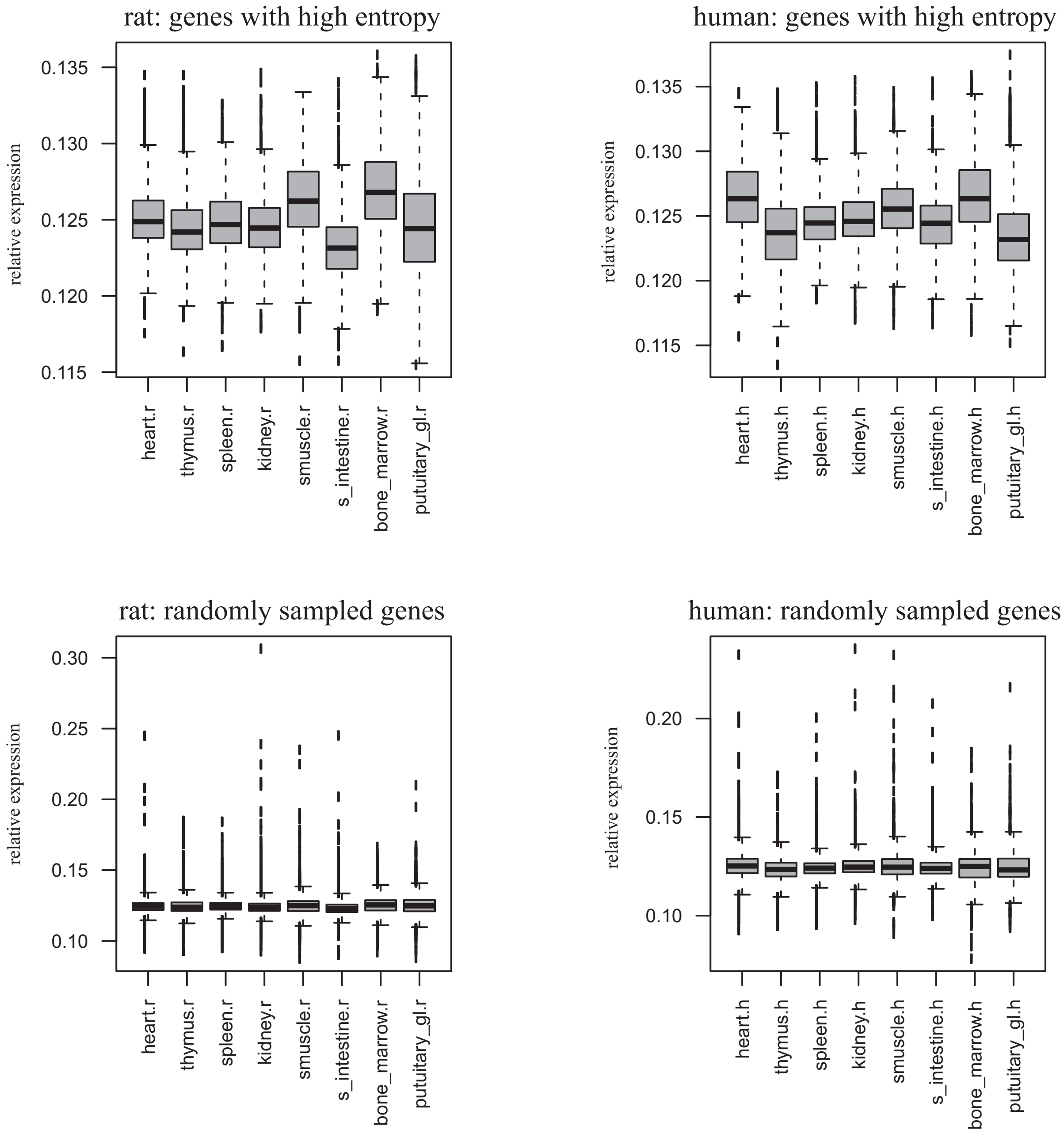

Figure S3. Expression profiles of 788 genes with the entropy in the upper quartile of mean entropy values (upper panel) and the same amount of randomly selected genes (lower panel).
